# Serum Soluble Urokinase-Type Plasminogen Activator Receptor Is Associated with Low Left Ventricular Ejection Fraction and Elevated Plasma Brain-Type Natriuretic Peptide Level

**DOI:** 10.1371/journal.pone.0170546

**Published:** 2017-01-30

**Authors:** Shu-ichi Fujita, Suguru Tanaka, Daichi Maeda, Hideaki Morita, Tomohiro Fujisaka, Yoshihiro Takeda, Takahide Ito, Nobukazu Ishizaka

**Affiliations:** Department of Cardiology, Osaka Medical College, Osaka, Japan; Scuola Superiore Sant'Anna, ITALY

## Abstract

**Background:**

Recent studies have suggested that soluble urokinase plasminogen activator receptor (suPAR), a biomarker of subclinical levels of inflammation, is significantly correlated with cardiovascular events.

**Purpose:**

We investigated the association between suPAR and left ventricular ejection fraction (LVEF), left ventricular mass index (LVMI), and plasma B-type natriuretic peptide (BNP) among cardiac inpatients.

**Methods and Results:**

In total, 242 patients (mean age 71.3 ± 9.8 years; 70 women) admitted to the cardiology department were enrolled in the study. suPAR was significantly correlated with LVEF (R = -0.24, P<0.001), LVMI (R = 0.16, P = 0.014) and BNP (R = 0.46, P<0.001). In logistic regression analysis, the highest suPAR tertile (> 3236 pg/mL) was associated with low LVEF (< 50%) and elevated BNP (> 300 pg/mL) with an odds ratio of 3.84 (95% confidence interval [CI], 1.22–12.1) and 5.36 (95% CI, 1.32–21.8), respectively, after adjusting for age, sex, log-transformed estimated glomerular filtration rate (log(eGFR)), C-reactive protein, and diuretic use. The association between suPAR and LVMI was not statistically significant. In multivariate receiver operating characteristic analysis, addition of log(suPAR) to the combination of age, sex, log(eGFR) and CRP incrementally improved the prediction of low LVEF (area under the curve [AUC], 0.827 to 0.852, P = 0.046) and BNP ≥ 300 pg/mL (AUC, 0.869 to 0.906; P = 0.029).

**Conclusions:**

suPAR was associated with low LVEF and elevated BNP, but not with left ventricular hypertrophy, independent of CRP, renal function, and diuretic use among cardiac inpatients who were not undergoing chronic hemodialysis.

## Introduction

The receptor for urokinase-type plasminogen activator (uPAR), a membrane-linked protein, may mediate immune and inflammatory activation and cancer cell progression [1,2,3,4]. Soluble uPAR (suPAR), which is formed by the cleavage and release of uPAR, has been gathering increasing attention owing to its potential as a biomarker for the presence or progression of various diseased conditions. For example, elevated suPAR levels have been shown to be associated with chronic kidney disease (CKD) and cardiovascular abnormalities, including coronary artery disease, early cardiac systolic and diastolic myocardial impairment, heart failure, and incident cardiovascular events [5,6,7,8,9,10,11,12,13]. Recent cohort studies showed that elevated suPAR levels were independently associated with incident chronic kidney disease, a decline in the renal function [14] and hospitalization due to impaired kidney function [15].

Despite the observed association between suPAR and several aspects of cardiovascular diseases, it remains unclear whether suPAR plays a causal role in the disease process, whether suPAR levels increase as a resultant of the disease process, or whether suPAR is a mere bystander [16].

Left ventricular systolic dysfunction and hypertrophy are presumed to have an association with low-grade inflammation [17,18,19]; however, only a few studies have investigated the possible association between cardiac function and left ventricular hypertrophy (LVH) and suPAR. In the current study, we retrospectively examined whether serum suPAR is associated with left ventricular ejection fraction (LVEF) and left ventricular mass (LVM) among cardiac inpatients who were not undergoing chronic hemodialysis.

## Methods

### Ethics statement

The current retrospective study was approved by the Ethics Committee at the Osaka Medical College and conducted in accordance with the Declaration of Helsinki. Written informed consent was obtained from all patients or their guardians.

### Study population

**Fig 1:**
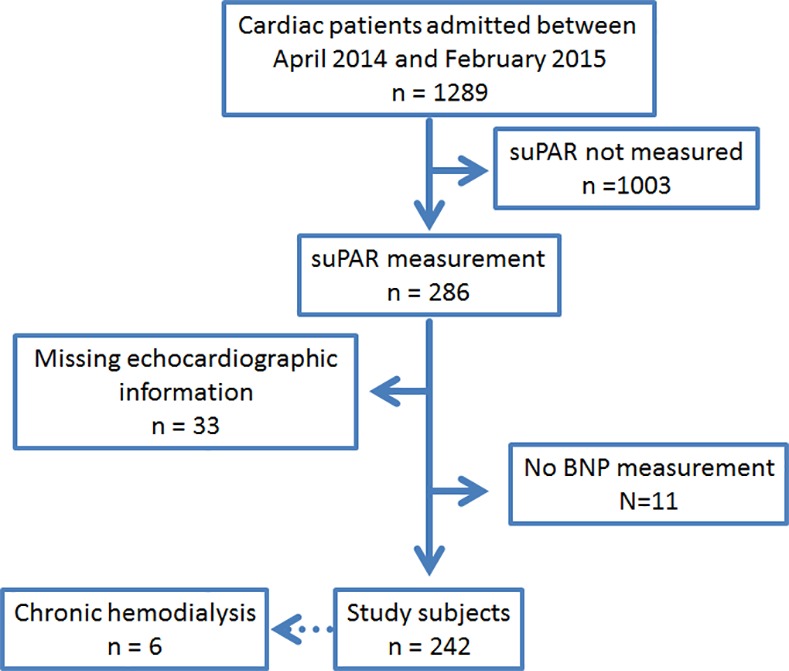
Flow diagram of the patient enrollment.

Between April 2014 and February 2015, 1289 patients were admitted to the cardiology department; among them, suPAR was measured in 286 consecutive patients after obtaining written informed consent. Of 286 patients, 33 for whom echocardiographic data were not sufficient for the current study, were excluded from the study population. A further 11 patients whose B-type natriuretic peptide (BNP) levels were not available were also excluded. Thus, 242 patients were enrolled as the study population, which included 6 patients who were undergoing chronic hemodialysis (Fig 1).

### Laboratory analysis

Blood samples were collected in the morning after an overnight fast. Aliquots of serum and plasma were immediately obtained and stored at -80 degrees until analysis. Serum levels of suPAR were measured by a kit (R&D Systems, Minneapolis, MN) according to the manufacturer’s instructions. High-sensitivity C-reactive protein (CRP) and BNP levels were measured by routine laboratory methods. The estimated glomerular filtration rate (eGFR) was calculated by the following Modification of Diet in Renal Disease equation for Japanese subjects: eGFR = 194 × (serum creatinine)^-1.094^ × (age)^-0.287^ (× 0.739, when female) [20].

### Echocardiography

Echocardiographic examinations were performed with a Vivid 7 Dimension equipped with a multi-frequency transducer (GE Healthcare, Vingmed, Norway). Left ventricular (LV) end-diastolic dimension (LVDd), interventricular septal thickness (IVST) and posterior wall thickness (PWT) were measured at end diastole. LV volumes were calculated by the modified Simpson method using the apical 4-chamber view. The LVEF was defined as low when < 50%. LVM was calculated by the formula proposed by Devereux et al. [21] with the following modification: 0.8 x 1.04 x [(LVDd + IVST + PWT)^3^—LVDd^3^] + 0.6 [22]. Body surface area (BSA) was calculated by using the following formula: (body weight)^0.425^ × (height)^0.725^ × 0.007184, and the LVM index (LVMI) was calculated as the ratio of LVM to BSA. When the LVMI was greater than 118 g/m^2^ (men) or 108 g/m^2^ (women), LV hypertrophy was defined as present [23].

### Statistical analysis

Baseline characteristics were assessed with standard descriptive statistics. Data were expressed either as mean ± standard deviation, number (percentage) or median and interquartile range (IQR). Spearman rank correlation test was used to assess the correlation between two variables. Multivariate logistic regression analysis was performed by SPSS statistics version 21.0 (IBM, Armonk, NY). Multivariate receiver operating characteristic (ROC) analysis was performed by STATA 12 (StataCorp LP, College Station, TX). For the multivariate analyses, only those who were not undergoing chronic hemodialysis were included, because eGFR was used as a covariate for the analysis.

## Results

### Patient characteristics

The demographic data, laboratory values, and echocardiographic parameters of the study subjects are summarized by suPAR tertile in Tables 1 and 2. Those with higher suPAR were older, but gender did not significantly differ significantly across the tertiles (Table 1). Moderate or severe heart failure (New York Heart Association functional class III or IV) was more than 10 times more prevalent among subjects in the highest suPAR tertile than among those in the lowest tertile. Patients with higher suPAR were more likely to be taking loop diuretics and thiazides.

**Table 1:** Demographic characteristics of the study patients by suPAR tertile.

		suPAR tertile						
Variables		Lowest (n = 80)		Middle (n = 81)		Highest (n = 81)		P value
suPAR range, pg/mL		513	-2021	2137	-3228	3236	-26131	
Age, years		68.3	±9.7	70.7	±9.3	75.0	±9.4	<0.001
Women, n (%)		22	(27.5)	15	(18.5)	23	(28.4)	0.274
Body mass index, kg/m^2^		23.8	±2.9	23.8	±3.5	22.8	±3.3	0.065
Systolic blood pressure, mmHg		130	±19	127	±17	123	±21	0.055
Pulse rate, bpm		72	±16	75	±15	75	±19	0.403
NYHA III/IV, n (%)		3	(4)	12	(15)	33	(41)	<0.001
*Smoking status*								
	Never, n (%)	37	(46.3)	21	(25.9)	30	(37.0)	0.032
	Former, n (%)	38	(47.5)	44	(54.3)	40	(49.4)	
	Current, n (%)	5	(6.3)	16	(19.8)	11	(13.6)	
Chronic hemodialysis, n (%)		1	(1.3)	0	(0.0)	5	(6.2)	0.028
*Cardiovascular disease*								
	Ischemic heart disease, n (%)	52	(65.0)	62	(76.5)	59	(72.8)	0.254
	Arrhythmic disease, n (%)	31	(38.8)	27	(33.3)	27	(33.3)	0.708
	Peripheral artery disease, n (%)	6	(7.5)	6	(7.4)	6	(7.4)	>0.99
	Valvular heart disease, n (%)	7	(8.8)	7	(8.6)	11	(13.6)	0.499
	Cardiomyopathy, n (%)	5	(6.3)	3	(3.7)	10	(12.3)	0.098
	Aneurysmal disease, n (%)	2	(2.5)	10	(12.3)	5	(6.2)	0.047
*Medication*								
	ACE inhibitors/ARB, n (%)	46	(57.5)	39	(48.1)	45	(55.6)	0.454
	Beta blockers, n (%)	37	(46.3)	34	(42.0)	38	(46.9)	0.791
	Calcium channel blockers, n (%)	37	(46.3)	36	(44.4)	33	(40.7)	0.772
	Diabetic medication, n (%)	16	(20.0)	18	(22.2)	31	(38.3)	0.017
	Statin, n (%)	47	(58.8)	38	(46.9)	33	(40.7)	0.068
	Loop, n (%)	7	(8.8)	9	(11.1)	45	(55.6)	<0.001
	Thiazide, n (%)	5	(6.3)	3	(3.7)	12	(14.8)	0.027
	Aldosterone antagonist, n (%)	5	(6.3)	7	(8.6)	24	(29.6)	<0.001

ACE, angiotensin converting enzyme; ARB, angiotensin receptor blocker.

**Table 2:** Laboratory and echocardiographic data of the study patients.

	suPAR tertile						
Variables	Lowest (n = 80)		Middle (n = 81)		Highest (n = 81)		P value
*Laboratory examination*							
White blood cell count, x10^3^/μL	5.5	(4.5–6.478)	6.2	(4.9–7.6)	5.8	(4.7–7.3)	0.075
Hemoglobin, g/dL	13.9	(12.7–14.9)	13.5	(12.3–14.8)	11.6	(10.6–13.0)	0.000
Platelet count, x10^3^/μL	20.0	(16.4–22.2)	18.7	(16.4–23.8)	18.4	(13.4–23.6)	0.370
Total cholesterol, mg/dL	184	(163–211)	180	(167–200)	151	(126–175)	0.000
Total protein, mg/dL	7.0	(6.7–7.3)	7.0	(6.6–7.4)	6.8	(6.3–7.3)	0.071
Albumin, mg/dL	4.2	(3.9–4.3)	4.0	(3.7–4.2)	3.7	(3.3–4.0)	0.000
ALT. U/L	18	(14–23)	19	(14–29)	17	(11–25)	0.163
Blood urea nitrogen*, mg/dL	16	(14–19)	16	(14–20)	21	(16–29)	0.000
Creatinine*, mg/dL	0.81	(0.67–0.95)	0.93	(0.76–1.11)	1.14	(0.89–1.51)	0.000
eGFR*, mL/min/1.73m^2^	52.6	(44.4–66.3)	46.3	(38.6–56.3)	35.6	(26.4–46.7)	0.000
B-type natriuretic peptide, pg/mL	32	(15–90)	54	(34–123)	107	(54–438)	0.000
Uric acid, mg/dL	5.7	(4.4–6.5)	5.8	(5.1–6.9)	6.2	(5.2–7.5)	0.016
C reactive protein, mg/dL	0.06	(0.04–0.15)	0.09	(0.04–0.34)	0.22	(0.10–0.99)	0.000
*Echocardiographic data*							
LVDd, mm	4.7	(4.4–5.1)	4.8	(4.4–5.2)	4.9	(4.5–5.6)	0.150
LVDs, mm	2.9	(2.6–3.5)	3.0	(2.8–3.8)	3.3	(2.8–4.3)	0.010
LVEF, %	63.0	(56.0–68.8)	60.0	(52.0–65.5)	56.0	(44.0–66.0)	0.003
LVMI, g/cm^2^	98.6	(80.2–113)	102	(86.0–127)	104	(85.8–128)	0.127

LVDd, left ventricular diastolic dimension; LVDs left ventricular systolic dimension; LVEF, left ventricular ejection fraction; LVMI, left ventricular mass index.

*For blood urea nitrogen, creatinine, and eGFR, values were analyzed only from those who were not undergoing chronic hemodialysis.

Patients with higher suPAR had greater CRP values and lower eGFR values (Table 2). Fifty-four patients (22%) had an eGFR of ≥ 60 mL/min/1.73m^2^. By Spearman correlation analysis, suPAR was significantly correlated with eGFR, age, hemoglobin serum albumin, BNP, and CRP with a coefficient of -0.495, 0.30, -0.45, -0.47, 0.46, and 0.40, respectively (all P < 0.001). In addition, suPAR also showed a significant correlation with LVEF (R = -0.24, P < 0.001), and with LVMI (R = 0.16, P = 0.014).

### Relationship between admission diagnosis and suPAR levels

Next, we examined whether certain cardiovascular condition on admission affected suPAR levels. Prevalence of neither acute myocardial infarction nor unstable angina pectoris did not significantly differ across the suPAR tertile. On the other hand, prevalence of worsening heart failure was significantly greater among the higher suPAR tertile. Prevalence of other admission diagnosis, including arrhythmic diseases, follow-up coronary angiography, stable angina pectoris, pre-operative cardiovascular screening for cardiovascular or non-cardiovascular surgery, aortic dissection, arteriosclerosis obliterans, or silent myocardial ischemia did not significantly differ across the suPAR tertile group (Table 3).

**Table 3:** Admission diagnosis for each suPAR tertile.

		suPAR tertiles						
Variables		Lowest (n = 80)		Middle (n = 81)		Highest (n = 81)		P value
*Admission diagnosis*								
	Acute myocardial infarction, n (%)	1	(1.3)	4	(4.9)	2	(2.5)	0.363
	Unstable angina pectoris, n (%)	7	(8.8)	7	(8.6)	3	(3.7)	0.358
	Worsening heart failure, n (%)	6	(7.5)	12	(14.8)	30	(37.0)	<0.001
	Stable angina pectoris, n (%)	11	(13.8)	7	(8.6)	6	(7.4)	0.362
	Arrhythmic diseases, n (%)	19	(23.8)	13	(16.0)	8	(9.9)	0.060
	Follow-up coronary angiography, n (%)	18	(22.5)	15	(18.5)	10	(12.3)	0.236
	Pre-operative screening before non-cardiovascular surgery n (%)	8	(10.0)	6	(7.4)	4	(4.9)	0.473
	Pre-operative screening before cardiovascular surgery n (%)	2	(2.5)	3	(3.7)	6	(7.4)	0.296
	Aortic dissection, n (%)	0	(0.0)	1	(1.2)	1	(1.2)	0.608
	Arteriosclerosis obliterans, n (%)	2	(2.5)	4	(4.9)	5	(6.2)	0.523
	Silent myocardial ischemia , n (%)	6	(7.5)	10	(12.3)	11	(13.6)	0.433

### Multivariate logistic regression analysis

By univariate logistic regression analysis, log(suPAR) was significantly associated with low LVEF (< 30%) and elevated BNP (≥ 300 pg/mL), but not with LVH (Table 4). The associations between suPAR and low LVEF and elevated BNP remained statistically significant after adjusting for sex, age, log(eGFR) (model 2), CRP (model 3), and diuretic use (model 4). When subjects with an eGFR of ≥ 60 mL/min/m^2^ (n = 54) and those with an eGFR of < 60 mL/min/m^2^ (n = 182) who were not undergoing hemodialysis were analyzed separately in model 3, the odds ratio of the highest suPAR tertile for low LVEF and elevated BNP was 6.95 (95% CI 1.91–25.35, P = 0.003) and 5.84 (95% CI 1.34–25.40, P = 0.019), respectively (eGFR of ≥ 60 mL/min/m^2^), and 7.38 (95% CI 0.76–71.45) and 23.20 (95% CI 0.98–551), respectively (eGFR of < 60 mL/min/m^2^).

**Table 4:** Logistic regression analysis for the association between suPAR and left ventricular mass, left ventricular ejection fraction, or B-type natriuretic peptide (BNP).

				suPAR tertile				
		Log(suPAR), per 1SD		Lowest	Middle		Highest	
		OR	95% CI	OR	OR	(95% CI)	OR	(95% CI)
Dependent variable: Low left ventricular ejection fraction								
	Model 1	2.07**	(1.47–2.93)	1 (ref)	2.15	(0.82–5.65)	6.00**	(2.43–14.8)
	Model 2	2.28**	(1.51–3.45)	1 (ref)	2.50	(0.91–6.86)	8.23**	(2.88–23.5)
	Model 3	2.06**	(1.33–3.19)	1 (ref)	2.09	(0.75–5.86)	6.48**	(2.21–19.0)
	Model 4	1.67*	(1.04–2.68)	1 (ref)	2.50	(0.83–7.52)	3.59*	(1.14–11.3)
	Model 5	1.14	(0.67–1.95)	1 (ref)	1.77	(0.54–5.83)	2.36	(0.62–8.98)
Dependent variable: Left ventricular hypertrophy								
	Model 1	1.13	(0.86–1.49)	1 (ref)	1.76	(0.89–3.50)	1.74	(0.87–3.50)
	Model 2	1.04	(0.75–1.44)	1 (ref)	1.89	(0.93–3.85)	1.61	(0.72–3.62)
	Model 3	0.99	(0.70–1.39)	1 (ref)	1.82	(0.89–3.73)	1.50	(0.65–3.43)
	Model 4	0.84	(0.58–1.20)	1 (ref)	1.96	(0.94–4.12)	0.90	(0.37–2.24)
	Model 5	0.72	(0.48–1.05)	1 (ref)	1.66	(0.78–3.54)	0.72	(0.28–1.87)
Dependent variable: Plasma BNP level ≥ 300 pg/mL								
	Model 1	3.51**	(2.23–5.51)	1 (ref)	2.05	(0.59–7.12)	9.75**	(3.21–29.6)
	Model 2	3.51**	(2.08–5.95)	1 (ref)	2.30	(0.62–8.51)	10.1**	(2.76–37.1)
	Model 3	3.35**	(1.95–5.76)	1 (ref)	2.04	(0.54–7.65)	8.51**	(2.27–32.0)
	Model 4	2.55**	(1.44–4.50)	1 (ref)	2.34	(0.57–9.67)	4.85*	(1.20–19.6)

Only those who were not undergoing chronic hemodialysis were included. Model 1, non-adjusted; model 2, adjusted for sex, age and log(eGFR); model 3, adjusted for variables used in model 2 plus CRP; model 4, adjusted for variables used in model 3 plus diuretic use; model 5, adjusted for the variables used in model 4 plus log(BNP).

* and ** indicate p<0.05 and P<0.01, respectively, for the 1 standard deviation increase for log(suPAR) and versus the lowest suPAR tertile for the middle and the highest suPAR tertiles. OR indicates odds ratio, CI indicates confidence interval, and ref indicates reference.

When we excluded the patients who were admitted due to the worsening heart failure or undergoing hemodialysis from the statistical analysis, it was found that, in model 4, middle and the highest suPAR was associated with low LVEF with an odds ratio of 1.64 (95% CI 0.44–6.14, P = 0.464) and 5.20 (95% CI 1.25–21.66 P = 0.023). In this analysis, 188 patients were analyzed. On the other hand, in this same model 4, neither the middle (odds ratio, 0.63; 95% CI 0.05–8.20, P = 0.721) or the highest (odds ratio, 1.80; 95% CI 0.20–15.81, P = 0.598) suPAR was not significantly associated with elevated BNP.

When we analyzed subjects with ischemic heart disease (n = 168) and those without (n = 188) separately in model 3, the odds ratio of the highest suPAR tertile for low LVEF and BNP ≥ 300 pg/mL was 15.5 (95% CI 3.33–72.2, P<0.001) and 9.66 (95% CI 0.94–99.5, P = 0.057), respectively (ischemic heart disease), and 1.77 (95% CI 0.30–10.30, P = 0.53) and 13.45 (95% CI 2.05–88.23, P = 0.007), respectively (no ischemic heart disease).

### Multivariate ROC analysis

**Fig 2:**
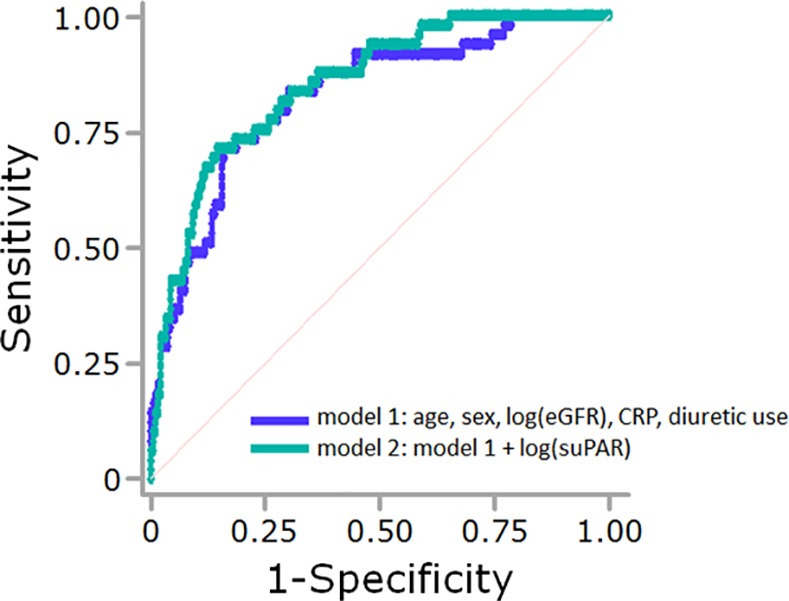
Receiver operating characteristic (ROC) analysis for the prediction of low left ventricular ejection fraction (LVEF). The purple line shows the ROC curve to predict low LVEF, for the combination of age, sex, log(eGFR), CRP, and diuretic use (model 1). The green line shows the ROC curve to predict low LVEF for model 1 plus log-transformed soluble urokinase-type plasminogen activator receptor (suPAR) (model 2). The area under the ROC curve was significantly greater in model 2 than in model 1 (0.827 versus 0.852, P = 0.046). In this analysis, only data from patients who were not undergoing chronic hemodialysis were included.

In multivariate ROC analysis, the area under the curve (AUC) to predict low LVEF, for the combination of age, sex, log(eGFR), CRP, and diuretic use was 0.827 (standard error [SE], 0.033), and further addition of log(suPAR) incrementally increased the prediction (AUC, 0.852; SE, 0.029, P = 0.046, Fig 2). The AUC to predict BNP ≥ 300 pg/mL, for the combination of age, sex, log(eGFR), CRP, and diuretic use was 0.869 (SE, 0.035), and further addition of log(suPAR) incrementally increased the prediction (AUC, 0.906; SE, 0.026, P = 0.029).

## Discussion

We herein demonstrated that suPAR was associated positively with LVEF and negatively with plasma BNP levels among cardiac patients. These associations were found to be independent of eGFR, CRP, and diuretic use. On the other hand, the association between suPAR and LVH was not significant after adjusting for various confounders.

Several previous studies have reported a relationship between suPAR and N-terminal prohormone BNP (NT-proBNP). For example, Kruger et al. reported that NT-proBNP was significantly associated with suPAR in black African subjects, but not in Caucasian subjects [13]. By analyzing data from the Malmö Diet and Cancer Study, a prospective cohort study conducted Malmö, Sweden, Borne et al. found that suPAR was significantly associated with increased plasma levels of NT-proBNP [12]. Although the mechanisms underlying the relationship between suPAR and BNP remain unclear, there are several possibilities. Subjects with increased suPAR may have enhanced systemic immune and inflammatory conditions [6,24] that may also be associated with the development of heart failure [25,26] and left ventricular function [27,28].

Mekonnen et al. also showed that coronary flow reserve was negatively associated with suPAR in patients with non-obstructive coronary artery disease [29]. Furthermore, Theilade et al. reported that, among patient with type 1 diabetes, subjects with increased suPAR were more likely to have a high degree of arterial stiffness [11]. These observations suggest that impaired coronary microcirculation and increased arterial stiffness may lead to left ventricular diastolic dysfunction [30,31]. In the current study, we could not assess the relationship between suPAR and diastolic dysfunction because of the small sample size-only 12 patients were judged to have diastolic dysfunction among patients with preserved LVEF (>50%, n = 193). This point should be investigated in the future studies.

We also found that patients with higher suPAR levels had lower LVEF (Table 2). To date, only a few studies have reported a relationship between suPAR and LVEF. By analyzing 318 patients with type 1 diabetes without known heart disease, Theilade et al. found that subjects with higher suPAR tended to have lower LVEF by univariate analysis, although this relationship was not significant after multivariate adjustment [11]. Theilade et al.’s population did not include patients with known heart disease or end-stage renal disease. On the other hand, our study population included both those who had more than moderately impaired renal function and those with ischemic heart disease. In the subgroup analysis in the current study, the association between high suPAR and low LVEF was more pronounced and significant among those with low eGFR and those with ischemic heart disease, respectively; therefore, the difference in the observation between Theilade et al.’s study and ours might be attributed to the different study population. Fewer studies seem to have investigated the relationship between suPAR and cardiac hypertrophy. In the above-mentioned study of Taheilade et al., suPAR did not have a significant association with LVMI after multivariate adjustment [11], in agreement with our study.

Interestingly, after excluding patients who were admitted due to the worsening heart failure from the analysis, the highest suPAR was still significantly associated with low LVEF with an odds ratio of 5.20 (95% CI 1.25–21.66 P = 0.023) after adjusting for age, sex, log(eGFR), CRP, and diuretic use. It was suggested, therefore, that suPAR may be independently associated with decreased LV function, although those who were admitted due to worsening heart failure had significantly higher suPAR levels compared with those who were admitted due to other reasons. It was shown by a recent experimental study that bone marrow Gr-1^lo^ immature myeloid cells may be responsible for the elevated, pathological levels of suPAR, and when these cells were transferred to healthy mice, it efficiently transmitted proteinuria when transferred to healthy animals [32]. Which cells were responsible for the increased suPAR among patients with low LVEF, and whether suPAR per ce play a role in promoting cardiac systolic dysfunction await further investigation.

Although suPAR was found to be associated with decreased LVEF and elevated of BNP independent of renal function and CRP, we do not propose routine measurement of suPAR in clinical practice. The utility of suPAR, in addition to its biomarker properties [16], may lie in its ability to increase our understanding of the pathogenesis of the observed cardiovascular abnormalities.

The current study has several limitations. First, owing to its cross-sectional nature, the study cannot provide information on the causal or resultant nature of the relationship. Second, the study subjects had various cardiovascular disorders because we enrolled patients who were admitted to the cardiology department. Although the data are relevant in real-world clinical practice; however, the possibility that the relationship between suPAR and left ventricular dysfunction differs according to certain specific cardiovascular conditions requires further evaluation. Third, the number of subjects with preserved LVEF was small; therefore, we could not examine the potential relationship between suPAR and cardiac diastolic dysfunction, which has been suggested in a previous study [11].

In conclusion, serum suPAR concentrations were associated with low LVEF (< 50%) and elevated plasma BNP (>300 pg/mL), but not with left ventricular hypertrophy among cardiac patients. The association between suPAR and low LVEF and elevated BNP remained significant after adjusting for age, sex, eGFR, CRP, and diuretic use. Whether suPAR represents a useful guiding biomarker for the treatment of cardiac dysfunction and heart failure, and whether it is involved in the progression of cardiac disorders await further investigation.
